# Commissioning and implementation of a pencil‐beam algorithm with a Lorentz correction as a secondary dose calculation algorithm for an Elekta Unity 1.5T MR linear accelerator

**DOI:** 10.1002/acm2.14590

**Published:** 2024-12-03

**Authors:** Sameer Taneja, Hesheng Wang, David L. Barbee, Paulina Galavis, Mario Serrano Sosa, David Byun, Michael Zelefsky, Ting Chen

**Affiliations:** ^1^ Department of Radiation Oncology New York University Langone Medical Center New York New York USA; ^2^ Department of Therapeutic Radiology Yale University School of Medicine New Haven Connecticut USA

**Keywords:** adaptive therapy, dose calculation, MR‐linac

## Abstract

**Purpose:**

To commission a beam model in ClearCalc (Radformation Inc.) for use as a secondary dose calculation algorithm and to implement its use into an adaptive workflow for an MR‐linear accelerator.

**Methods:**

A beam model was developed using commissioning data for an Elekta Unity MR‐linear accelerator and entered into ClearCalc. The beam model consisted of absolute dose calculation settings, output factors, percent depth‐dose (PDD) curves, mutli‐leaf collimator (MLC) transmission and dose leaf gap error, and cryostat corrections. Beam profiles were hard‐coded by the manufacturer into the beam model and were compared with Monaco‐derived profiles. The beam model was tested by comparing point doses in a homogenous phantom obtained through measurements using an ionization chamber in water, Monaco, and ClearCalc for various field sizes, source‐surface distances (SSDs), and point locations. Additional testing including point dose verification for test plans using a heterogeneous phantom and patient plans. Post clinical implementation, performance of ClearCalc was evaluated for the first 41 patients treated, which included 215 adaptive plans.

**Results:**

PDDs generated using ClearCalc fell within 1.2% of measurements. Field profile comparison between ClearCalc and Monaco showed an average pass rate of 98% using a 3%/3 mm gamma criteria. Measured cryostat corrections used in the beam model showed a maximum deviation from unity of 1.4%. Point dose and field monitor units (MUs) comparisons in a homogenous phantom (*N* = 22), heterogeneous phantoms (*N* = 22), and patient plans (*N* = 57) all passed with a threshold of 5%/5MU. Clinically, ClearCalc was implemented as a physics check post adaptive planning completed prior to beam delivery. Point dose and field MUs showed good agreement at a 5%/5MU threshold for prostate stereotactic body radiation therapy (SBRT), pelvic lymph nodes, rectum, and prostate and lymph node plans.

**Discussion:**

This work demonstrated commissioning and clinical implementation of ClearCalc into an adaptive planning workflow. No primary or adaptive plan failures were reported with proper beam model testing.

## 1 INTRODUCTION

Independent dose calculations^1^ are part of comprehensive quality assurance (QA) programs for external beam radiation therapy. These safety barriers limit potential errors in planning and delivery of radiation prior to patient treatment^2^ by confirming, via a dose recalculation, that the treatment fields and plans yield radiation doses within tolerance of that calculated by the original treatment planning system. This is done through independent dose and monitor unit (MU) number comparisons (dose/MU). The American Association of Physicists in Medicine (AAPM) provide guidelines and recommendations on accurately implementing secondary dose/MU calculation engines into clinical use, specifically in task group (TG) numbers 71.^3^ and more recently 219.^4^ Commissioning of the secondary dose/MU calculation includes performing tests in accordance with recommendations from AAPM TG‐53.^5^ and MPPG5a.,^6^ as well as performing a comparison between measurements and dose calculations at a series of machine parameters and point locations based on TG‐219.^4^

The use of secondary dose/MU calculation algorithms are particularly important in online adaptive treatments, such as those frequently performed in magnetic resonance imaging (MRI)‐guided radiotherapy using MR‐linear accelerators. The online adaptive workflow of these treatments involves generating a new plan for each fraction based on an initial MRI scan of patient setup and internal anatomy. With the patient on the couch during adaptive planning, it is not feasible to perform pre‐treatment patient specific QA using conventional measurement‐based methods. As a result, secondary dose/MU calculations provide the only independent verification of calculated dose of the adaptive plan prior to delivery.

Secondary dose/MU calculations for MR linear accelerators present additional complexity in comparison to traditional linear accelerator calculations due to the presence of the magnetic field affecting generated charged particles via the Lorentz force and the increased source to central axis geometry required due to the combination of a magnet and ring‐based accelerator. Multiple groups have developed in‐house and explored commercial secondary dose/MU calculation software utilizing various dose calculation algorithms. The accuracy of RadCalc (Lifeline Software Inc., Tyler, TX), which utilizes a Clarkson integration technique, was studied for use with a 0.35T ViewRay MRidian^7^ (ViewRay Inc, Oakland, CA) and a 1.5T Elekta Unity (Elekta, Stockholm, Sweden).^8^ MR‐linear accelerators. Various collapsed cone methods using commercial OnCentra (Elekta),^9^, ^10^ Mobius 3D (Varian Medical Systems Inc., Palo Alto, CA)^11^ and RayStation (Raysearch Laboratories, Stockholm, Sweden)^12^ have been implemented with Unity accelerators. Yang et al.^13^ used an analytical anisotropic algorithm (AAA) in Eclipse (Varian Medical Systems) for secondary dose/MU checks for a Unity

The purpose of this work is to evaluate ClearCalc (CA), manufactured by Radformation Inc. (New York, NY), which utilizes a finite‐size pencil beam (FSPB) algorithm with a Lorentz correction for magnetic field for a 1.5T Elekta Unity MR‐linear accelerator. Beam modeling and validation are described in this study. To the authors’ knowledge, previous work has not been presented on the ClearCalc software and the author aims to provide a reference for other clinics in ClearCalc implementation.

## 2 METHODS

For an in‐depth description of the Elekta Unity MR‐linear accelerator, Raaymakers et al. presented an initial proof of concept and a review of the first patients treated in 2009^14^ and 2017^9^, respectively. The Unity pairs a 1.5T MRI (Philips Marlin, Philips Healthcare, Amsterdam, Netherlands) with a 7‐MV flattening filter free (FFF) photon linear accelerator with the capabilities of step‐and‐shoot intensity modulated radiation therapy (IMRT) and three‐dimensional (3D) conformal treatments. The beam is shaped using jaws with a maximum field size of (57.4 × 22) cm^2^ in the *x*‐ and *y*‐planes, respectively, and mutli‐leaf collimator (MLC) consisting of 160 leaves. The fixed dose rate is 425 MU/min at the source‐isocenter distance of 143.5 cm.

All commissioning and implementation presented in this work was performed using ClearCalc v.2.4.6. This version of ClearCalc provided one significant change to the beam model from the initial version, v2.2.9: a hardcoded cryostat correction that is specific to the author's machine based on commissioning measurements, as opposed to a manufacturer‐defined cryostat correction.

### 2.1 Beam model

An Elekta Unity beam model was generated using dosimetric data obtained during linear accelerator commissioning. The following section describes measurements and simulation that comprised the beam model.

Absolute dose calibration to 1 cGy per MU in water was completed in an source‐axis distance (SAD) configuration using a reference field size of 10 × 10 cm^2^ at an source‐surface distance (SSD) of 138.5 cm and a depth of 5 cm. Calibration measurements were completed using a PTW TN30013 (PTW, Freiburg, Germany) Farmer‐type ionization chamber using methods outlined by TRS 398.^15^ and correction factors outlined by recent studies.^16^, ^17^, ^18^

Output factors, in the form of a total scatter factor (S_c,p_),^19^ were measured using an SSD of 133.5 cm and at a depth of 10 cm. Measurements were completed in a BEAMSCAN MR (PTW, Freiburg, Germany) water phantom using a Semiflex 3D ionization chamber (Model No. 31031, active volume: 0.07 cm^3^, PTW Dosimetry) for field sizes larger than 10 × 10 cm^2^ and a microdiamond detector (Model No. 60019, active volume: 0.004 mm^3^, PTW Dosimetry) for field sizes smaller than 10 × 10 cm^2,^. Field sizes of equivalent squares of 2 × 2 cm^2^, 3 × 3 cm^2^, 5 × 5 cm^2^, 10 × 10 cm^2^, 15 × 15 cm^2^, 22 × 22 cm^2^, 29 × 29 cm^2^, and 34 × 34 cm^2^ were added to the ClearCalc beam model.

Percent depth dose (PDD) curves were measured at an SSD of 133.5 cm using a Semiflex 3D ionization chamber for equivalent field sizes ranging from 0.5 × 0.5 cm^2^ to 30.2 × 30.2 cm^2^ at a gantry angle of 0°. The maximum depth of measurement was 13 cm. This work used a method for inputting PDDs published by Graves et al.,^8^ in which PDDs used for the ClearCalc model were generated using Monaco Treatment Planning System (Elekta, Stockholm, Sweden) equipped with a commissioned beam model^8^ for the Unity machine. PDDs were simulated with a grid size of 1 mm and an uncertainty of 0.3% for equivalent‐square field sizes of 2, 3, 4, 7, 10, 20, 22, 25.4, 27.2, and 30.2 cm^2^. In order to evaluate accuracy of the Monaco‐determined PDDs, a comparison of simulated and measured PDDs was performed at depths of 5 cm, 10 cm, and 13 cm for equivalent‐square field size ranging from 2 to 22 cm^2^.

MLC transmission and dosimetric leaf gap error were also added into the beam model. Transmission was measured using an Exradin A1SLMR (Standard Imaging Inc., Middleton WI) ionization chamber at a depth of 5 cm in solid water, orientated perpendicular to MLC motion, and set at an SSD of 138.5 cm. 2000 MU were delivered separately with the Y1 jaw closed and with the Y2 jaw closed. The chamber reading with the MLCs closed was compared with an open field reading, and the average transmission between the Y1 jaw and Y2 jaw was added to the beam model. As Monaco utilizes various parameters when defining the MLC model and ClearCalc utilizes a single DLG value, DLG value in the ClearCalc model was optimized by comparing dose differences with Monaco plans for phantom and clinical reference CT‐based and adaptive MR‐based plans. Values were determined by the manufacturer and from work by Tsuneda et al.^20^ Point doses for identical point locations were tested for 12 primary and 10 adaptive plans using dosimetric leaf gap errors of 0.01, 0.02, 0.1, 0.15, 0.20, and 1.0 cm, which spans the accepted range of DLG values able to be entered into ClearCalc. The smallest difference between ClearCalc and Monaco was set as the DLG value in the beam model.

The cryostat is a think pipe containing liquid helium required for the design of the linear accelerator. It requires a correction to characterize the output of the machine as a function of gantry angle due to non‐uniform attenuation resulting from the cryostat component. The cryostat attenuation was measured using a PTW TN30013 Farmer‐type ionization chamber using a 10 × 10 cm^2^ open beam. A measurement was made every 2°, with the exception of gantry angles 8°–18° to avoid penetrating the cryostat pipe in that direction. All readings were normalized relative to the reading at gantry angle 0°. This machine‐specific correction was added into the ClearCalc beam model.

In addition to user‐set parameters in the ClearCalc beam model, field profiles generated by Radformation were set in the ClearCalc beam model specifically for the Unity machine. These profiles were determined using beam profiles for a 6 MV FFF energy with an updated alpha factor to account for difference in attenuation due to energy and Lorentz force. As the user cannot adjust the beam profiles in the ClearCalc beam model, this study evaluated the accuracy of the beam profiles inClearCalc for a small (2 × 2 cm^2^), medium (10 × 10 cm^2^), and large field (50 × 22 cm^2^).

### 2.2 Model validation

The ClearCalc beam model generated for the Unity MR‐linear accelerator was benchmarked using methods outlined by AAPM TG‐219 and MPPG5a, which recommend performing commissioning tests for a secondary MU calculation similar to those of a conventional treatment planning system. The beam model was validated using a comparison of point dose measurements for a homogenous phantom, for a heterogeneous phantom, and for pre‐clinical plans.

Dose/MU measurements were compared in a homogenous water phantom using three methods: ionization chamber measurements in water, calculated using the treatment planning system, and calculated using ClearCalc. Dose/MUs were determined at SSDs of 133.5, 138.5, and 143.5 cm, field sizes of 2 × 2 cm^2^, 3 × 3 cm^2^, 4 × 4 cm^2^, 10 × 10 cm^2^, 5 × 20 cm^2^, 20 × 5 cm^2^, and 50 × 22 cm^2^, and various dose points both on‐ and off‐axis. Ionization chamber measurements were performed in the PTW BEAMSCAN MR water phantom using a Semiflex ionization chamber. 100 MU were delivered to the detector at the point of measurement and the ionization chamber charge reading was corrected for temperature and pressure. An N_DW_ calibration factor, and beam quality (k_Q_) transfer factor corrected for the magnetic field were applied to the corrected reading to convert collected charge to dose. Dose/MU was also determined in the Monaco treatment planning system using a homogenous phantom QA plan with a dose grid spacing of 3 mm and an uncertainty in calculation of 1%. The QA plan was exported to ClearCalc for analysis. The percent differences calculated between the three dose/MU calculation methods had a tolerance of homogenous beams with high dose and low gradient settings set to 5%.

Measurements performed in a homogenous phantom followed guidelines from TG‐219 but also fulfilled tests recommended by MPPG5a. This included, test 5.2: dose in test plan versus reference calibration condition, test 5.3: TPS data versus commissioning data, test 5.4: small MLC‐shaped field, test 5.5: large MLC‐shaped field with extensive blocking, test 5.6: off‐axis MLC shaped field, and test 5.7: asymmetric field at minimal anticipated SSD. Test 5.8, which tested a 10 × 10 cm^2^ field at oblique incidence (> 20°) was not measured but compared between Monaco and ClearCalc.

**FIGURE 1:**
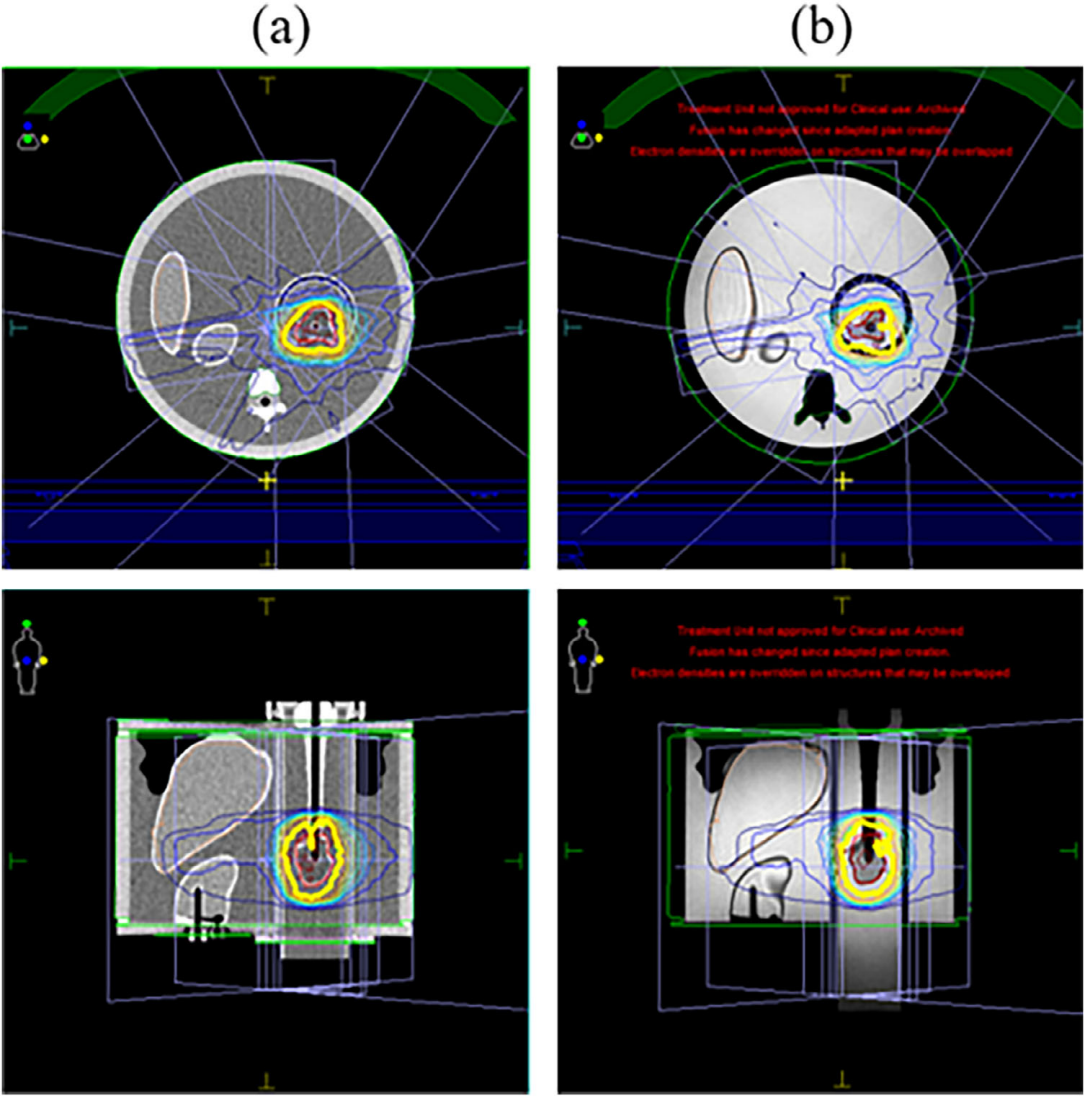
Axial and Coronal views of the Zeus 0087Z heterogenous phantom for (a) a primary plan using a CT and (b) an adaptive plan using an MR scanned on the unity.

Point dose comparisons were calculated between treatment planning system and ClearCalc were tested for a homogenous phantom, a Zeus model 008Z (Sun Nuclear Corporation) used without motion, for abdominal treatments. A primary plan was generated on a planning CT dataset of the phantom, with the intended dose of 66 Gy in 30 fractions to a contoured liver CTV (Figure 1). Seven fields were used to cover 95% of the CTV volume to receive the prescribed dose. Adaptive plans were generated on Unity‐generated MR scans and planned using an adaptive workflow. Although there was no change in anatomy, electron density was generated from contours on the MR scan. These tests were performed on a primary plan that used a CT simulation of the phantom as the primary imaging and multiple adaptive plans that used Unity‐generated MR scans for planning. Point doses and individual field dose averages were reported.

A non‐clinical plan was generated on a heterogenous dataset to evaluate the accuracy of ClearCalc at various locations in accordance with TG‐219. Calculation points were set in tissue, superficial tissue, tissue‐lung interfaces, lung, and bone as well as set in uniform dose or gradient regions and overall point doses comparisons were reported. Finally, a set of non‐clinical plans were developed for various treatment sites and dose schema and tested prior to clinical implementation. Plans were completed on the MR imaging set acquired from the Unity on volunteers. Plans were generated to represent clinical plans and calculations were performed as part of the plan QA to mimic clinical workflow. Plan point doses and individual field MUs were calculated and the difference between ClearCalc and Monaco were reported.

### 2.3 Clinical integration

ClearCalc was implemented into an adaptive treatment workflow for the Unity MR‐linear accelerator. In addition to using ClearCalc for a secondary MU check for the primary plan, policy and procedures were written to perform ClearCalc calculations by physics prior to treatment for all adaptive plans.

A total of 41 patients with 215 adaptive plans were treated from October 2023 to July 2024 using the adaptive plan workflow on the Unity. Plans were primarily prostate (*N* = 27) but also included pelvic nodes (*N* = 7), rectum due to prostatic recurrence (*N* = 1), and prostate and lymph nodes (*N* = 8). The MUs necessary to achieve field's measured dose were calculated in ClearCalc and compared to the treatment planning system for each field individually with a tolerance of 5% or 5 MUs. In addition, the overall dose calculation was performed at appropriate calculation points and had a tolerance of 5% or 5 cGy. The user can generate points or use pre‐determined points in ClearCalc for these calculations.

## 3 RESULTS

### 3.1 Beam model

**FIGURE 2:**
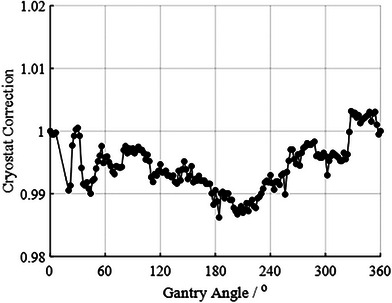
Cryostat correction showing correction factor as a function of gantry angle, and added as part of the ClearCalc beam model.

All machine‐specific and calibration settings and values that were added to the Unity beam model in ClearCalc are shown in Table 1. An absolute dose calibration of 1 cGy per MU was completed at a reference field size of 100 mm, an SSD of 138.5 cm, and a calibration depth of 5 cm. The MLC transmission factor and DLG was measured to be 0.0055 and 0.01 cm, respectively. PDDs were simulated using the Monaco TPS due to limitations in a measurement depth of 13 cm. A comparison of PDD values at obtainable depths of 5, 10, and 13 cm was performed for equivalent square field sizes of 2, 3, 5, 10, and 22 cm and showed a maximum deviation of 1.1% between measured and TPS‐calculated values (Table 2). Figure 2 shows a plot of the measured cryostat correction factor input into the ClearCalc model as a function of gantry angle, normalized to a gantry angle of 0°. The maximum and minimum correction factor was 0.3% and 1.1%, respectively. MLC leaf transmission was set to 0.055. The DLG optimization is shown in Table 3, in which dose/MU differences between Monaco and ClearCalc were completed for DLG settings of 0, 0.01, 0.02, 0.1, 0.15, 0.2, and 1 cm. DLG of 0.01 cm showed the best agreement, and was used in the model.

**TABLE 1:** Machine specific and calibration settings in ClearCalc beam model.

Parameter	Value
Machine energies	7X‐FFF
Absolute dose reference field size (mm)	100
Absolute dose calibration Source to plane distance (mm)	1385
Absolute dose calibration depth (mm)	50
Reference dose at calibration depth (Gy)	1
Reference MU at calibration depth (MU)	100
MLC transmission factor	0.0055
Dosimetric leaf gap (cm)	0.010

Abbreviations: FFF, flattening filter free; MU, monitor unit.

**TABLE 2:** Comparison of measured PDDs and Monaco‐derived PDDs added to the ClearCalc beam model.

	5 cm depth			10 cm depth			13 cm depth		
Field (mm)	Measured	ClearCalc	Difference (%)	Measured	ClearCalc	Difference (%)	Measured	ClearCalc	Difference (%)
20	0.835	0.830	0.522	0.640	0.634	0.640	0.539	0.528	1.126
30	0.850	0.839	1.145	0.646	0.649	−0.338	0.547	0.538	0.869
50	0.861	0.857	0.390	0.672	0.674	−0.180	0.570	0.563	0.689
100	0.883	0.880	0.327	0.704	0.708	−0.353	0.606	0.602	0.364
220	0.885	0.885	0.029	0.724	0.724	−0.027	0.634	0.629	0.478

Abbreviation: PPDs, percent depth‐doses.

**TABLE 3:** Average dose difference [%] between the treatment planning system and ClearCalc for primary, adaptive, and overall for various leaf gap error settings in the ClearCalc model.

Gap error (cm)	Dose difference—Primary (%)	Dose difference—Adaptive (%)	Average dose difference (%)
0.0	1.62	1.55	1.59
0.01	1.43	1.61	1.51
0.02	1.49	1.70	1.58
0.10	1.56	2.22	1.86
0.15	1.73	2.65	2.15
0.20	2.08	3.29	2.63
1.00	9.81	9.76	9.79

**FIGURE 3:**
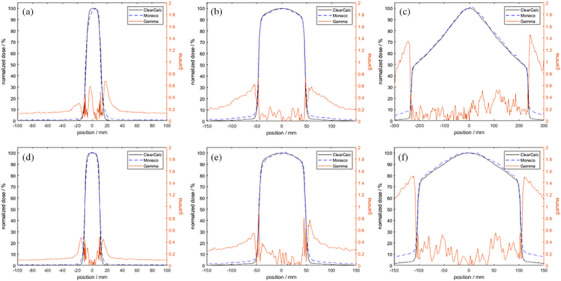
Beam profile comparison between ClearCalc and Monaco, along with gamma analysis results with gamma criteria of 5%/5  mm for cross plane profiles for (a) 2 × 2 cm^2^, (b) 10 × 10 cm^2^, and (c) 50 × 22 cm^2^ field sizes and for in plane profiles for (d) 2 × 2 cm^2^, (e) 10 × 10 cm^2^, and (f) 50 × 22 cm^2^ field sizes.

Dose plan comparisons between Monaco and ClearCalc are shown in Figure 3 for small (2 × 2 cm^2^), medium (10 × 10 cm^2^), and large (50 × 22 cm^2^) field sizes and for cross‐plane and in‐plane directions. All points in‐field, defined by the 50% dose field edge, fell within the MPPG5a test 5.2 recommended 2% threshold. A 2‐dimensional global gamma analysis was performed using 5% dose difference or 5 mm distance‐to‐agreement (DTA) criteria and with a 10% dose threshold. The gamma pass rate was 100% at these settings for all profiles. The majority of the deviation between Monaco and ClearCalc profiles was below the 10% dose threshold.

### 3.2 Model validation

A total of 27‐point doses were used for validation, in which dose was compared between ionization chamber measurements in water, Monaco, and ClearCalc for clinical SSDs, field sizes, and for both on‐axis and off‐axis points (Table 4). It was found that all points passed with the maximum deviation between ClearCalc and Monaco of 3.5% and the maximum deviation between ClearCalc and ionization chamber measurements of 2.7%. Although AAPM TG‐219 recommended these measurements, they also fulfilled specific guidelines from MPPG5a, which test numbers 5.2–5.7 were specifically denoted as subscripts in the “TPS‐CC” column. Test 5.8, which compared a 10 × 10 cm^2^ field at an oblique incidence of > 20° was not measured, but was planned in Monaco and compared with ClearCalc and found to match within 0.06%.

**TABLE 4:** Point dose verification comparing measurement (Meas)‐, Monaco (TPS)‐, and ClearCalc (CA)‐determined point dose values for various SSDs, field sizes, and point positions.

SSD (cm)	Jaw position (X,Y)	Dose point (x,y,z)	Measured (cGy)	TPS (cGy)	ClearCalc (cGy)	Meas‐TPS (cGy)	Meas‐CA (cGy)	TPS‐CA (cGy)
133.5	10,10	0,0,10	86.5	86.1	85.6	0.5	1.0	0.65^.^3
133.5	5,20	0,0,5	105.6	105.7	105.0	−0.1	0.6	0.75^.^7
133.5	5,20	0,5,5	96.3	97.0	95.6	−0.7	0.8	1.5
133.5	5,20	0,0,13	72.5	72.1	72.1	0.5	0.5	0.0
133.5	20,5	0,0,5	105.8	105.4	105.5	0.4	0.3	−0.1
133.5	20,5	−5,0,5	96.5	96.3	95.8	0.2	0.7	0.5
133.5	20,5	0,0,13	72.6	72.1	71.9	0.7	1.0	0.3
133.5	4,4	0,0,5	100.2	99.9	99.4	0.3	0.8	0.55^.^4
133.5	4,4	0,0,13	66.3	65.5	64.6	1.2	2.6	1.4
133.5	50,22	0,0,13	87.6	87.1	87.3	0.6	0.4	−0.2^5.5^
133.5	50,22	12.5,0,13	65.4	64.9	63.7	0.8	2.6	1.9
133.5	50,22	0,5,13	80.9	80.6	79.3	0.4	2.0	1.6
133.5	50,22	−12.5,0,13	64.7	64.4	63.9	0.4	1.2	0.8
133.5	50,22	0,‐5,13	81.8	80.7	79.3	1.4	3.1	1.8
138.5	10,10	0,0,5	100.5	100.2	100	0.3	0.5	0.25^.^2
138.5	[‐10,20],10	15,0,5	65.8	64.8	66.8	1.5	−1.5	−3.0^5.6^
138.5	[‐10,20],10	16.5,0,8	55.5	54.9	56.7	1.1	−2.2	−3.2
138.5	[20,‐10],10	−15,0,5	65.1	64.5	66.3	0.9	−1.8	−2.8
138.5	[20,‐10],10	−16.5,0,8	54.8	54.3	56.4	1.0	−2.8	−3.8
143.5	2,2	0,0,1	94.7	95.6	95.7	−0.9	−1.0	−0.1
143.5	2,2	0,0,3	87.8	89.8	86.7	−2.3	1.3	3.5
143.5	3,3	0,0,1	100.1	99.4	97.4	0.7	2.7	2.0
143.5	50,22	0,0,1	116.7	115.8	117.7	0.8	−0.9	−1.6
143.5	50,22	0,5,3	103.3	104.0	105.2	−0.6	−1.8	−1.1
143.5	50,22	0,‐5,3	104.4	104.0	105.1	0.3	−0.7	−1.1
143.5	50,22	12.5,0,3	82.8	82.7	83.5	0.2	−0.8	−1.0
143.5	50,22	−12.5,5,3	79.2	79.2	80.4	0.1	−1.4	−1.5

Abbreviation: SSDs, source‐surface distances.

The performance of the ClearCalc model was subsequently tested on a heterogeneous phantom for both a primary plan using a planning CT image set and for an adaptive plan using an MR‐scan from the Unity. Differences between ClearCalc and Monaco for the plan point dose was 1.2% and 2.0% with an average (standard deviation) individual field MUs of 1.5% (2.2%) and 1.5% (1.5%) for the primary plan and adaptive plan, respectively. All individual field MU differences fell within the set clinical tolerance of 5%/5 MUs.

Dose points in various interfaces, material, and isodose lines in a single heterogenous dataset are shown in Table 5. All points passed a 5%/5 MU tolerance. In addition, preclinical plans were tested prior to using ClearCalc clinically. Table 6 shows the ClearCalc results for clinically deliverable plans for prostate, prostate + dominant intraprostatic legion (DIL), partial brain, pelvic nodes, prostate and pelvic nodes, and liver on a combination of CT and MR images. Individual field MU differences and point doses are presented. All fields and dose points passed at a 5%/5MU tolerance.

**TABLE 5:** Dose differences between ClearCalc and Monaco for various point locations in a heterogenous non‐clinical dataset.

Material	Dose distribution	Dose difference (%)
Tissue	Uniform	0.57
Tissue—Superficial	Gradient	3.79
Tissue‐Lung	Uniform	3.05
Tissue‐Lung	Gradient	4.67
Lung	Uniform	3.83
Lung	Gradient	4.02
Bone	Uniform	4.83
Bone	Gradient	4.50

**TABLE 6:** Pre‐clinical plan comparison between ClearCalc and Monaco.

						Individual fields		
Site	Total dose (cGy)	Fractions	Field No.	Imaging	% Difference (%)	Mean (%)	Max (%)	Min (%)
Prostate	4000	5	11	CT	−0.8	−0.9	3.7	−3.4
Prostate	4000	5	11	MRI	2.9	−0.9	1.8	4.0
Prostate + DIL	4500	5	11	CT	−0.2	1.2	3.8	−1.8
Prostate + DIL	4500	5	11	MRI	2.3	−1.7	4.1	−4.5
Partial brain	6000	30	11	CT	1.0	−1.8	0.3	−3.9
Partial brain	6000	30	11	MRI	0.7	0.0	2.0	−4.5
Prostate and pelvic nodes	2500	5	15	CT	1.5	−0.6	3.2	−3.7
Prostate and pelvic nodes	2500	5	15	MRI	3.9	−0.9	4.8	−3.9
Liver	6000	3	18	CT	3.0	−0.8	4.1	−4.2

Abbreviations: DIL, dominant intraprostatic legion; MRI, magnetic resonance imaging.

### 3.3 Clinical integration

Post commissioning, ClearCalc was implemented into the planning and adaptive treatment planning workflow for the Unity MR‐linear accelerator. During planning, ClearCalc was used by the physicist as a secondary MU check software during the physics check. In the adaptive workflow used during each treatment fraction, the adaptive plan was generated by the physics team and approved by the physician. Upon approval but prior to beam on, the images, structure set, and individual beam doses were exported to a folder on a shared drive. ClearCalc configuration was set to parse the folder and perform a secondary MU calculation. Once the secondary MU calculation was complete and indicated a passing result, physics gave the go‐ahead to treat.

The Unity linear accelerator went live with treatment of primarily prostate cancers with a plan to expand to other disease sites. The performance of ClearCalc's agreement with Monaco was evaluated retrospectively for the first 41 patients and 215 adaptive plans. Of these plans, 27 were prostate stereotactic body radiation therapy (SBRT) plans with dose schemas of (1) 40 Gy in 5 fractions with a simultaneous subvolume boost of additional 5 Gy to the DIL, (2) 40 Gy in 5 fractions to a prostate target volume, and (3) 25 Gy in 5 fractions to a prostate target volume after an initial brachytherapy boost. In addition, seven cases treated the pelvic oligometastatic lymph nodes with dose schemes of 27 Gy in 3 fractions, 36 Gy in 6 fractions, and 30 Gy in 5 fractions. One case was treated to the rectum with a dose scheme of 35 Gy in 5 fractions. Finally, eight cases were treated to the prostate and lymph nodes with dose schemes of 25 Gy in 5 fractions.

**FIGURE 4:**
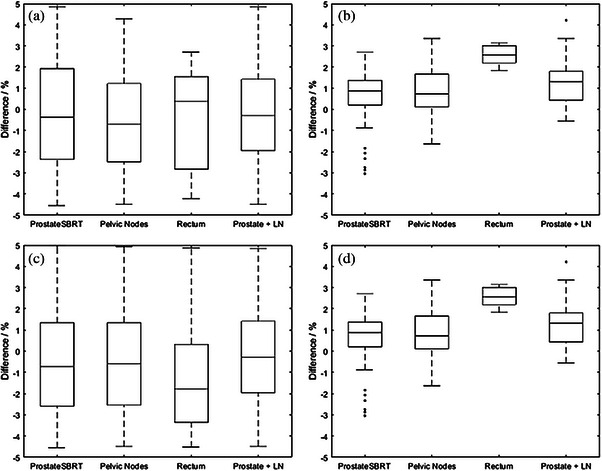
Boxplots showing the differences between ClearCalc and Monaco for clinical plans, separated into (a) individual fields for primary plans, (b) dose points for primary plans, (c) individual fields for adaptive plans, and (c) dose points for adaptive plans.

For each reference and adaptive plan, ClearCalc's secondary dose/MU check for individual field MUs and overall plan point dose with a tolerance of 5% / 5MUs showed no failures. Figure 4 shows differences between ClearCalc and Monaco for field MUs and dose point comparisons for each treatment site for both primary plan and adaptive plans. For prostate SBRT treatments, the average (standard deviation) dose difference was 0.29% (1.3%) and 0.76% (1.0%) and treatment field difference was −0.2% (2.5%) and −0.5% (2.5%) for primary and adaptive plans, respectively. For pelvic node treatments, the average (standard deviation) dose difference was 1.0% (1.4%) and 0.9% (1.3%) and treatment field difference was −0.5% (2.3%) and −0.4% (2.5%) for reference and adaptive plans, respectively. For the single rectum treatment, the average (standard deviation) dose difference was 2.4% and 2.6% (0.5%) and treatment field difference was −0.4% (2.4%) and −1.2% (2.5%) for primary and adaptive plans, respectively. Finally, for prostate and lymph node treatments, the average (standard deviation) dose difference was 2.0% (0.8%) and 1.3% (1.0%) and treatment field difference was −0.3% (2.4%) and −0.3% (2.4%) for primary and adaptive plans, respectively.

## 4 DISCUSSION

The purpose of this work was to share the author's experience with commissioning and benchmarking a beam model in ClearCalc for use as secondary dose calculations on an MR‐linear accelerator and to evaluate its clinical performance. This work follows clinical implementations guides from previous work for MR‐linear accelerators using various programs,^7^, ^8^, ^9^, ^10^, ^11^, ^12^ and particularly based on guidelines from the AAPM.^4^, ^6^ ClearCalc is unique in that it implements a direct Lorentz correction factor to account for dosimetric impacts from the magnetic field. The accuracy of this method was seen particularly in the good agreement between ClearCalc and a benchmarked model of Monaco across all tests in this work. There are limitations that are associated with the FSPB dose calculation method that ClearCalc utilizes as the algorithm balances speed of calculation with dosimetric accuracy. The approximation of dose deposition from the algorithm is limited in its ability to model charged particles passing in and out of material interfaces. Table 5 shows the evaluation of ClearCalc's performance at tougher geometries, including interfaces of bony anatomy and lung and at the edge of the dose distribution where the gradient is larger. ClearCalc showed agreement with Monaco under the 5%/5 MU threshold. In addition, the limitations in the model were also present in small fields, demonstrated in the deviations in beam profiles, particularly in the penumbra, between Monaco and ClearCalc for the 2 × 2 cm^2^ cross‐plane profile (Figure 2a). Although there were deviations in the beam profile, the homogenous dose regions showed good agreement and overall dose/MU calculations passed all commissioning tests for primary and adaptive treatment planning. This makes it a useful tool for secondary MU calculations. Graves et al.^8^ also reported on these deviations in beam profiles and limitations with the Clarkson integration technique algorithm and also concluded that these deviations showed little impact on the ability to provide a secondary dose calculation using an appropriate calculation point.

Radformation offers a standard beam model for a Unity MR‐linear accelerator. During initial implementation, it was found that although the beam model worked for the majority of test points in the homogenous water phantom (Table 4), there were specific points, particularly at small field sizes, that exceeded the 5% threshold. Ultimately, the authors used the institution‐specific beam model developed for Monaco supplemented with additional measurements. As a result, it is recommended to test the manufacturer's standard beam model prior to implementing it into clinical use.

When entering a beam model in ClearCalc's beam configuration workspace, there are few inputs open to the user. The user enters the parameters included in Table 1 and PDDs, but parameters like beam profiles, Sc and Sp individual values, MLC physical information, and the cryostat are all included in the model but not available for adjustment. The model generated with clinical beam data from commissioning was robust and passed the clinical benchmark tests, but had they not, it may have warranted an investigation into the hard‐coded data. An example of this is when ClearCalc was upgraded to version 2.4.6 from version 2.2.9, in which one adjustment was the addition of an institutionally unique cryostat correction instead of a manufacturer‐determined cryostat correction. The difference between the two cryostat corrections varied by up to 1.4%. Using the same gantry angles for reference and adaptive clinical plans, up to a 0.9% improvement was seen after the adjustment.

During the adaptive plan workflow, the patient is on the treatment couch and the speed of calculation is important for a secondary calculation algorithm. Although no timing data was obtained in this work, it was found that ClearCalc performed the calculation rather quickly and that the largest time sink was the plan export from an online Monaco workstation to the ClearCalc server. Once the required data is sent to the parsed ClearCalc folder, the specific treatment plan for the present date shows up in the ClearCalc interface immediately. The user can then click on the plan and the calculation is performed. Two methods that the author's implemented to speed up this process is to routinely archive the ClearCalc dicom folder to move all files for finished treatments and to only export the body, bones, immobilization, couch and coil structures from the plan. It was found that this amount of data in the export was sufficiently accurate calculations. However, more structure exports would yield a more accurate synthetic CT for calculation but it was noticed that during the commissioning, some calculations had trouble handling structures with complicated topologies and overlaps. In addition, the external designation from a structure was found to be critical in defining the overall CT boundaries.

One aspect that speeds up the calculation is that ClearCalc automatically finds appropriate points of interest saving the user from manually finding points. ClearCalc first checks the primary reference point that is denoted in planning, followed by the isocenter and then the centroid of target volumes. If these points fail to meet criteria, ClearCalc will find a point that is > 3 mm from tissue interfaces, > 1 cm from all field edges, within the 90% prescribed dose region (can be loosened to 70%), and a maximum point exposure by the field. In addition, the user can manually define points of interest for both plan point dose and individual field dose. The process of finding points and performing calculations were similar for all types of plans, from more complex and larger fields in pelvic and lymph node plans to less modulated pelvic node plans. It was noticed that the main difference in time between overall analysis was from the export.

Prior to performing dose/MU calculation, ClearCalc allows the user to edit the structure layers for the calculation. As only the body, bones, and support structures were exported for calculation, the structure layering did not impact the calculation. However, if additional structures were exported, it would be recommended to use the same structure layering as used for the plan. For adaptive plans, there is no reference CT data, which prompts the user to use the structure set with HU overrides. As a result, exporting the external structure is critical for accurate calculation.

It is recommended to perform extensive testing prior to clinical implementation to test the robustness of the beam model. For example, a series of failing clinical plans were able to isolate an incorrect SSD value in the initial beam model's calibration setting, which was set to an SSD of 133.5 cm. Based on homogenous and heterogeneous phantom testing and pre‐clinical plans, it was found that the tolerance of 5% or 5 MUs was appropriate based on the level of calculation precision. Clinically, no failing plans have been identified.

## 5 CONCLUSION

Independent dose/MU calculations are critical as a second check for the treatment planning system accuracy and is especially critical for high dose SBRT treatments. This work commissioned and verified a beam model of a Unity MR‐linear accelerator in ClearCalc, a third‐party secondary dose/MU calculation software. After extensive testing on various phantoms and pre‐clinical plans, ClearCalc was implemented into adaptive clinical workflow, where it has performed highly with accurate and speedy calculations. This work aims to guide other intuitions implementing a secondary dose/MU calculation software in performing clinically oriented tests to evaluate performance while also fulfilling AAPM guidelines.

## AUTHOR CONTRIBUTIONS

All authors have contributed to this work. Sameer Taneja is the corresponding author and Ting Chen is the senior author.

## CONFLICT OF INTEREST STATEMENT

The authors declare no conflicts of interest.
